# Genetic counselling and personalised risk assessment in the Australian pancreatic cancer screening program

**DOI:** 10.1186/s13053-019-0129-1

**Published:** 2019-10-23

**Authors:** Tanya Dwarte, Skye McKay, Amber Johns, Katherine Tucker, Allan D. Spigelman, David Williams, Alina Stoita

**Affiliations:** 1 Australian Pancreatic Cancer Genome Initiative, Garvan Institute of Medical Research, Darlinghurst, NSW Australia; 2 Hereditary Cancer Centre, Prince of Wales Hospital, Randwick, NSW Australia; 3 University of New South Wales, Prince of Wales Clinical School, Sydney, NSW Australia; 4 Department of Gastroenterology, St Vincent’s Hospital, Darlinghurst, NSW Australia; 5 Cancer Genetics Unit, The Kinghorn Cancer Centre, St Vincent’s Hospital, Darlinghurst, NSW Australia; 6 St Vincent’s Clinical School, University of New South Wales, Sydney, NSW Australia

**Keywords:** Pancreatic cancer screening, Endoscopic ultrasound, PancPRO, Genetic counselling, Personalised risk assessment

## Abstract

**Background:**

Pancreatic cancer (PC) is an aggressive disease with a dismal 5-year survival rate. Surveillance of high-risk individuals is hoped to improve survival outcomes by detection of precursor lesions or early-stage malignancy.

**Methods:**

Since 2011, a national high-risk cohort recruited through St Vincent’s Hospital, Sydney, has undergone prospective PC screening incorporating annual endoscopic ultrasound, formal genetic counselling and mutation analysis as appropriate. PancPRO, a Bayesian PC risk assessment model, was used to estimate 5-year and lifetime PC risks for familial pancreatic cancer (FPC) participants and this was compared to their perceived chance of pancreatic and other cancers. Genetic counselling guidelines were developed to improve consistency. Follow-up questionnaires were used to assess the role of genetic counselling and testing.

**Results:**

We describe the Australian PC screening program design and recruitment strategy and the results of the first 102 individuals who have completed at least one-year of follow-up. Seventy-nine participants met the FPC criteria (≥ two first-degree relatives affected), 22 individuals had both a *BRCA2* pathogenic variant and a close relative with PC and one had a clinical diagnosis of Peutz-Jeghers syndrome. Participants reported a high perceived chance of developing PC regardless of their genetic testing status. PancPRO reported FPC participants’ mean 5-year and lifetime PC risks as 1.81% (range 0.2–3.2%) and 10.17% (range 2.4–14.4%), respectively. Participants’ perceived PC chance did not correlate with their PancPRO 5-year (r = − 0.17, *p* = 0.128) and lifetime PC risks (r = 0.19, *p* = 0.091). Two-thirds felt that current genetic testing would help them, and 91% of tested participants were glad to have undergone genetic testing. Overall, 79% of participants found genetic counselling to be helpful, and 88% reported they would recommend counselling to their relatives.

**Conclusions:**

Participants reported multiple benefits of genetic counselling and testing but continue to seek greater clarification about their individual PC risk. Extension of PancPRO is required to enable personalised PC risk assessment for all high-risk sub-groups. More detailed discussion of PC risk for *BRCA2* pathogenic variant carriers, providing a written summary in all cases and a plan for genetics review were identified as areas for improvement.

## Background

Pancreatic cancer (PC) is an aggressive and devastating disease. Over 3000 new PC diagnoses were predicted in Australia in 2018 [1]. Whilst this represents only 2.4% of new cancers, PC disproportionately accounts for 6.2% of cancer deaths [1], a trend that is mirrored worldwide [2]. Typically, patients present with advanced disease, making surgical interventions impossible and treatments ineffective [3]. Most PC patients die within months of diagnosis [4], with only 7.7% surviving 5 years [1]. Early identification remains the only successful approach to longer term survival [5].

PC is most commonly sporadic, however 5–10% of PC cases are due to a genetic predisposition [6, 7]. This includes pathogenic variants in cancer predisposition genes, e.g. *BRCA2* and *PALB2*; Lynch Syndrome, Peutz-Jeghers Syndrome (PJS), Familial Atypical Multiple Mole Melanoma and Hereditary Pancreatitis [7, 8]. In addition, many families have apparently autosomal dominant transmission of PC without an identified pathogenic variant (“Familial Pancreatic Cancer (FPC))” [9]. The lifetime risk of PC within FPC families varies according to the number of affected first degree relatives (FDR), with a relative risk of 6.4 (lifetime risk 8–12%) for 2FDR and a relative risk of 32 (lifetime risk 40%) for ≥3FDR [10, 11]. Although screening for PC is not feasible nor recommended in general population due to the low incidence of PC, there is increasing evidence high-risk individuals ((HRI)) may benefit from PC screening in a research setting [12].. The Cancer of the Pancreas Screening (CAPS) consortium recommends PC screening for individuals with > 5% lifetime risk of PC [13]. The rationale of screening asymptomatic HRI is to diagnose precursor lesions or early PC when still resectable and hence, potentially curable to improve survival. Endoscopic ultrasound (EUS) and magnetic resonance imaging (MRI) are the screening methods of choice [13, 14]. EUS is minimally invasive and studies report high sensitivity and specificity for detecting PC lesions less than 2 cm and can also collect biopsy samples [14]. A systematic review showed that screening in HRI led to a higher diagnostic rate of pancreatic tumours than in controls (34% vs 7.2%, *p* < 0.001) [15]. PC screening resulted in a significantly higher curative resection rate (60% versus 25%, *p* = 0.001) and a significantly longer median survival time (14.5 month versus 4 months, p < 0.001) [15]. A recently published long-term (16-year) follow-up study of HRI reported that 90% of tumours identified during their PC surveillance program were resectable with a median time from baseline screening to PC diagnosis of 4.8 years and 85% of patients with resected cancers were alive at 3 years [16]. Importantly, these benefits occur in the absence of psychological harms [17, 18].

The Australian PC screening program was established in 2011 at St Vincent’s Hospital, Sydney, in collaboration with the CAPS consortium. To identify high-risk individuals, the Australian Familial Pancreatic Cancer Cohort (AFPaCC) was established under the Australian Pancreatic Cancer Genome Initiative (APGI) [19] as a registry and biorepository of individuals and families with a strong family history of PC. Efforts by the APGI to profile the genomic landscape of PC, including a subset of FPC patients, have been reported elsewhere [20–23].

The importance of genetic counselling for cancer predisposition syndromes is well recognised, with numerous studies reporting patient satisfaction, improved risk perception and better psychosocial outcomes [24–28]. Genetic counselling for heterogenous conditions like PC can be challenging as there are both strong environmental and inherited factors, and up until recently a lack of standardised genetic testing options [12, 29]. Increasingly, genomic testing is offered to PC patients, thus the need to ensure genetic counselling practices are effective and evidence-based.

The provision of genetic counselling for PC susceptibility is further hindered by insufficient data to estimate an individual’s PC risk. Several studies have assessed PC incidence within families with *BRCA2* pathogenic variants demonstrating a 4.4–5.9 relative risk (lifetime risk 5–8%), with a slightly higher risk for males [30–32]. Currently, no risk assessment models exist to clarify the PC risk in *BRCA2* carriers with and without a family history of PC. For FPC families, Wang et al. [33] developed a Bayesian risk estimation model, PancPRO, to quantify an individuals’ PC risk based on family history. This program considers the number of PC affected relatives, their age at diagnosis, and the age of unaffected relatives to calculate the likelihood of an individual harbouring a “PC susceptibility gene” and their 5-year and lifetime risks of developing PC [33]. PancPRO is freely available as part of the CancerGene software from http://www4.utsouthwestern.edu/breasthealth/cagene. PancPRO has been evaluated in only a small number of actual and theoretical PC populations [34, 35], and further validation is required.

## Methods

The aims of this study are: (1) to describe the recruitment and management of an Australian high-risk PC cohort; (2) to ascertain participant feedback regarding genetic counselling and testing for PC; and (3) to assess the utility of PancPRO to estimate PC risk for FPC participants and to compare it with their perceived cancer risks.

### Participants

Recruitment was achieved in one of five ways: 1) self-referral via the AFPaCC website; 2) identification as an FDR of an affected individual(s) meeting FPC criteria on the AFPaCC registry; 3) Clinical genetics service/Familial Cancer Clinic (FCC) referral; 4) General Practitioner (GP) or specialist referral; or 5) referral from collaborative cancer organisations (e.g. PanCare, KconFab, Avner Pancreatic Cancer Foundation). While most FCC referrals were prospective, ethics approvals were also sought at key Sydney clinics to allow for retrospective contact of eligible individuals. Participants were assessed to determine eligibility (Additional file 1) by the clinical research coordinator (TD and SM). Where possible, confirmation of PC diagnosis was obtained by pathology reports or death certificates.

### PC screening protocol

**Fig. 1:**
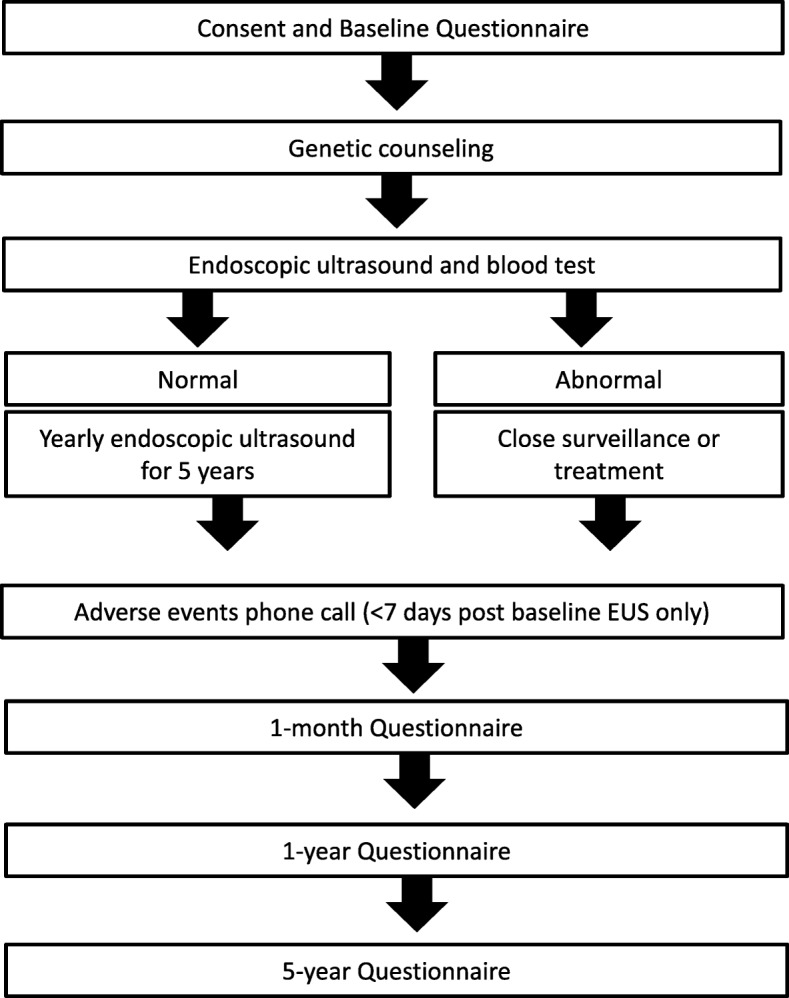
Summary of the PC screening protocol

All eligible participants provided written consent and completed a baseline personal and family health history questionnaire (Fig. 1). Formal genetic counselling was a prerequisite, with most participants having individual counselling. If a participant was referred from an FCC, this prerequisite was assumed to have been met. One interstate family attended a group counselling appointment, however after negative feedback this was discontinued. To facilitate consistent counselling, genetic counselling guidelines were designed and distributed to FCCs (Additional file 2).

Participants were scheduled for baseline EUS and blood tests, including CA-19.9, C-reactive protein, blood glucose and Macrophage Inhibitory Cytokine 1 (MIC-1), previously identified as a possible marker for PC [36]. Within a week of the baseline EUS, participants received a follow-up phone call to assess their experiences, note any complications and answer further queries. The screening program gastroenterologist allocated participants to continue annual EUS screening (normal EUS) or more intense surveillance by MRI or EUS at 3 or 6 months (abnormal EUS), depending on findings. Follow-up questionnaires are completed one-month, 1-year and 5-years post baseline EUS to assess both the value of genetic counselling and determine the psychological impact of screening.

### Instrumentation

Baseline and follow-up questionnaires were modified from the CAPS protocol with permission. A 5-point Likert scale was administered at baseline to assess perceived chance of developing PC and other cancer(s) (1 = much below others to 5 = much above others). At one-month post baseline EUS a follow-up questionnaire using a total of 19 statements with a 5-point Likert scale (1 = strongly agree to 5 = strongly disagree) and two open-ended questions, was administered to assess the overall experience of genetic counselling. Validated psychological assessment scales were also administered at each timepoint; these will be reported separately.

### Assessment of PC risk

For each participant within the FPC cohort, family history from the baseline questionnaire was assessed using PancPRO (CancerGene Version 6, The Bayes Mendel Group, UT Southwestern Medical Center, Dallas, USA), to calculate their likelihood of carrying a PC susceptibility gene and their 5-year and lifetime PC risk. PancPRO was an internal tool, with the participants and genetic counsellors blinded to the results. As PancPRO does not model the impact of pathogenic variants or clinical diagnosis, PancPRO was not used to generate a personalised PC risk estimate for other screening participants.

### Data analysis

Data analysis was performed using IBM SPSS Statistics for Windows (Version 25.0. Armonk, NY). Basic descriptive statistics were generated for participant demographics and genetic counselling responses. Spearman’s Rho was used to assess correlations between perceived PC and other cancer(s) chance scores with all continuous variables (e.g. age at enrolment, number of FDR/total number of PC affected relatives, 5-year and lifetime PC risk etc.). Mann-Whitney U tests were also used to assess differences with perceived PC and other cancer(s) chance scores with binomial data (e.g. gender, depression status and personal cancer history).

## Results

**Fig. 2:**
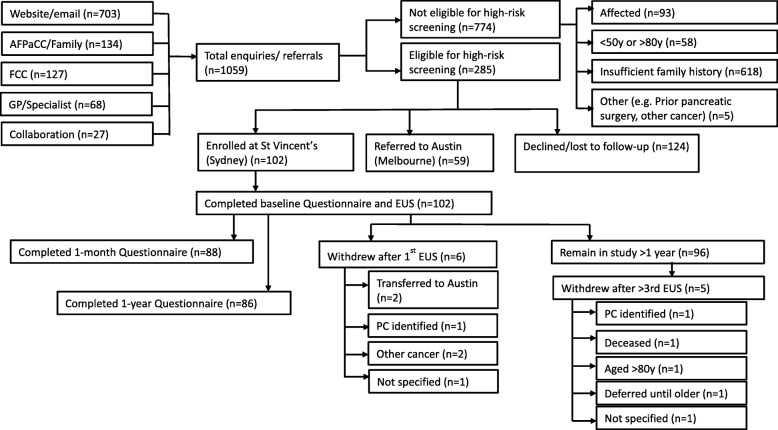
Diagram of participant eligibility, recruitment and retention for the PC screening program

From September 2011 to March 2017, 1059 individuals residing across all Australian states contacted or were referred to AFPaCC, based on their family history of PC. Seven hundred and seventy-four individuals were not eligible for the high-risk screening program at St Vincent’s Hospital (SVH), Sydney, as they were either affected by PC, had insufficient family history, or met one or more of the exclusion criteria. The remaining 285 eligible individuals were provided with a participant information and consent form and baseline questionnaire. Surprisingly,124 individuals (44%) did not proceed with enrolment.. Some were “passive decliners” meaning they did not return their recruitment paperwork (the coordinator made two repeat contact attempts). Anecdotally, factors influencing dropout were distance and expense of travel, psychosocial factors (e.g. caring for or grieving the loss of a family member with PC), a lack of perceived benefit and the amount of paperwork required. A second PC screening site was established at the Austin Hospital in Melbourne, Victoria, in 2014 and 59 Victorian participants were referred to this additional site and are not described further in this paper. At the time of analysis, a total of 102 participants provided written consent, completed the baseline questionnaire, commenced EUS screening at SVH and have completed at least 1 year of follow-up. Eleven participants (11%) have transferred to Melbourne or withdrawn from the screening protocol (Fig. 2).

### Participant demographics

Prospective data was analysed for the first 102 participants (comprising of 62 families). The average number of EUS was 3.3 but all had at least one EUS at the time of analysis. The mean age was 56 years (35–78 years) at enrolment, 69% were female, 99% were Caucasian with 12% reporting Ashkenazi Jewish ancestry (Table 1). The mean age of youngest PC diagnosis in the family was 55.2 years (range 21–84 years). As 56 participants (55%) were referred from an FCC, their genetic counselling took place prior to study recruitment (“off-protocol”) with the remaining 46 participants (45%) being referred to an FCC for genetic counselling after study recruitment (“on-protocol”).

**Table 1:** Demographics and genetic testing results

Criteria		Number (%)
Gender	Male	32 (31.4)
	Female	70 (68.6)
Ethnicity	White/Caucasian (non-Jewish)	89 (87.3)
	White/Caucasian (Jewish)	12 (11.8)
	Asian	1 (1.0)
Age		Mean (Range)
	Overall	56 (35-78y)
	Male	57 (37-78y)
	Female	56 (35-72y)
Family history	1 FDR (plus 1 or more SDR)	38 (37.3)
	2 FDR (+/− 1 or more SDR)	30 (29.4)
	3 FDR (+/− 1 or more SDR)	11 (10.8)
	*BRCA2* (plus 1 or more relative with PC)	22 (21.6)
	Clinical diagnosis of PJS	1 (1.0)
Genetic testing and previous malignancy		
Not tested	**Total**	**31 (30.4)**
(including PJS participant)	None	30 (29.4)
	Breast	1 (1.0)
No mutation identified	**Total**	**49 (48.0)**
(Self or close affected relative)	Nil cancer	42 (41.2)
	Thyroid	1 (1.0)
	Breast	5 (4.9)
	Melanoma	1 (1.0)
*BRCA2* carrier	**Total**	**22 (21.6)**
	Nil cancer	10 (9.8)
	Breast	5 (4.9)
	Breast – Bilateral	4 (3.9)
	Prostate	1 (1.0)
	Prostate/Brain tumour	1 (1.0)
	Melanoma	1 (1.0)
Smoking status^a^	Never smoked	56 (55.4)
	Previous smoker	41 (40.6)
	Current smoker	4 (4.0)
Alcohol consumption^b^	Non-drinker	17 (17.0)
	Social drinker	19 (19.0)
	Weekly drinker	36 (36.0)
	Daily drinker	28 (28.0)

^a^ one data point not declared; ^b^ two data points not declared

### Genetic testing and PC risk assessment using PancPRO

Seventy-one participants (or their close affected relative) underwent genetic investigation(s) during their genetic counselling appointment(s). Of these, 22 (14 females and eight males) were identified as a *BRCA2* pathogenic variant carrier. One female *BRCA2* carrier also had a second pathogenic variant detected in *BRCA1*. The family history of 79 participants met the classification of FPC. Genetic testing was performed on an affected relative (*n* = 40) or the PC screening participant (*n* = 9), of which 47 had no mutation detected in either *BRCA1/2* (*n* = 42), *MLH1/PMS2* (*n* = 7), *STK11* (*n* = 6), or showed preserved staining of the mismatch repair proteins using IHC (*n* = 10). Two additional participants were found to have a variant of uncertain significance (VUS) in *BRCA2* (considered no mutation identified). A single participant had a clinical diagnosis of PJS but declined genetic testing.

**Fig. 3:**
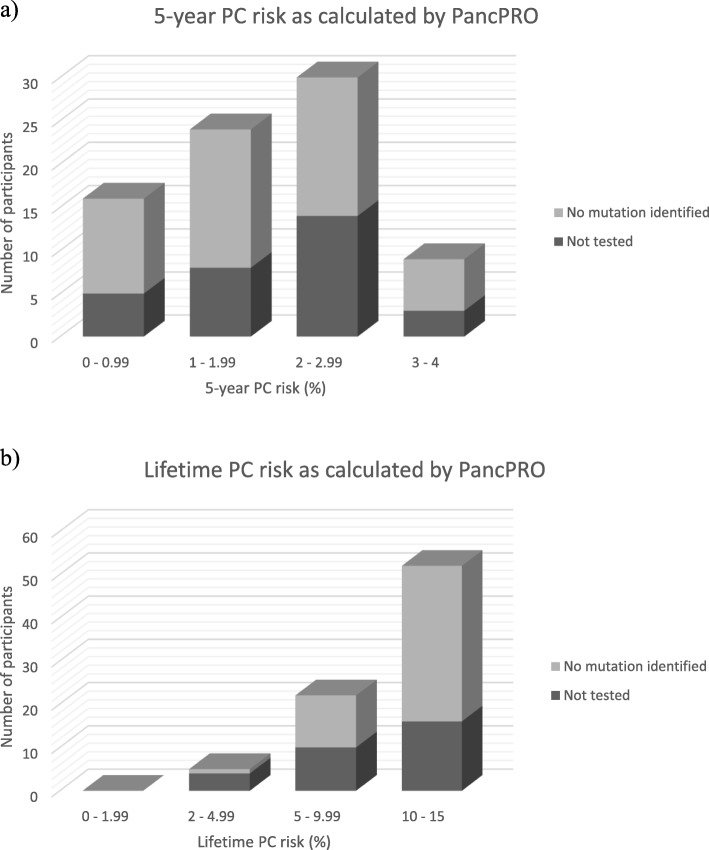
**a**) The 5-year and **b**) lifetime PC risk of the FPC cohort as calculated by PancPRO. Each bar represents the number of participants within the specified range of PC risk. The corresponding genetic testing status (no mutation identified or not tested) is indicated for each participant

In the FPC cohort (*n* = 79), PancPRO estimated a mean 5-year PC risk of 1.81% (range 0.2–3.2%) and a mean lifetime PC risk of 10.17% (range 2.4–14.4%) (Fig. 3). The mean probability of having a hypothetical PC susceptibility gene was 0.431 (43.1%, range 0.047–0.501). There was a significant moderate positive correlation with the total number of affected FDR/second degree relative (SDR) and probability of a PC susceptibility gene (r = 0.52, *p* < 0.001). The PancPRO risk estimates of our FPC cohort were further categorised based on number of affected relatives (Table 2). The mean lifetime PC risks calculated by PancPRO for individuals with 1FDR, 2FDR and 3FDR were 10.9% (range 3.7–14.4%), 9.5% (range 2.7–13.2%) and 9.7% (range 2.4–14.2%), respectively.

**Table 2:** Comparison of PancPRO estimates for the PC screening cohort based on participants’ family history (number of FDR and SDR affected with PC), pathogenic variant status or clinical diagnosis

Family History	Probability PC susceptibility gene			5-year PC risk			Lifetime PC risk			n
	Mean	Lowest	Highest	Mean	Lowest	Highest	Mean	Lowest	Highest	
1FDR (overall)	**0.431**	**0.159**	**0.497**	**0.014**	**0.003**	**0.032**	**0.109**	**0.037**	**0.144**	**38**
1FDR + 1SDR^a^	0.311	0.159	0.469	0.012	0.02	0.025	0.081	0.05	0.122	8
1FDR + 2SDR	0.463	0.267	0.497	0.011	0.003	0.027	0.119	0.037	0.144	19
1FDR + 3SDR	0.456	0.409	0.482	0.019	0.013	0.032	0.112	0.071	0.128	7
1FDR + 4SDR^b^	0.473	0.456	0.484	0.024	0.017	0.032	0.108	0.081	0.130	4
2FDR (overall)	**0.427**	**0.047**	**0.507**	**0.022**	**0.009**	**0.032**	**0.095**	**0.027**	**0.132**	**30**
2FDR	0.409	0.047	0.479	0.023	0.010	0.032	0.088	0.031	0.128	14
2FDR + 1SDR	0.427	0.195	0.483	0.023	0.009	0.032	0.095	0.027	0.125	12
2FDR + 3SDR	0.491	0.477	0.507	0.019	0.010	0.029	0.121	0.104	0.132	4
3FDR (overall)	**0.445**	**0.320**	**0.501**	**0.020**	**0.005**	**0.032**	**0.097**	**0.024**	**0.142**	**11**
3FDR	0.393	0.320	0.497	0.022	0.013	0.032	0.073	0.024	0.130	5
3FDR + 1SDR^b^	0.489	0.465	0.501	0.018	0.005	0.032	0.117	0.084	0.142	6

^a^Exception made due to early-onset PC +/− personal history of early-onset cancer; ^b^ representing one family

### Perceived chance of developing PC and other cancers

**Fig. 4:**
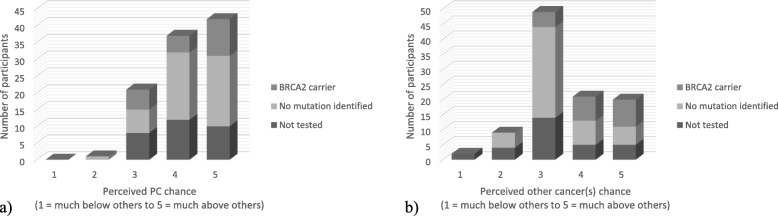
**a**) Participants’ perceived chance of developing PC and **b**) Participants’ perceived chance of developing other cancers. For each graph, bars represent the number of participants who selected the specified Likert value (1 = much below others, 3 = neutral, 5 = much above others) to indicate their extent of perceived chance. The corresponding genetic testing status is also shown for each participant

Participants’ perceived chance scores for developing PC and other cancer(s) are shown in Fig. 4. Participants reported a significantly higher perceived chance of developing PC compared to other cancer(s) (mean = 4.18 vs 3.47, *p* < 0.01, *n* = 102). There was no significant difference in perceived chance of developing PC between *BRCA2* carriers and those with either no mutation identified or those not tested (mean = 4.16 vs 4.23, *p* = 0.679). Appropriately, *BRCA2* carriers had a significantly higher perceived chance of developing other cancer(s) compared to the FPC cohort (mean = 4.18 vs 3.28, *p* < 0.001). Similarly, participants with a personal history of cancer had a significantly higher perceived chance of developing other cancer(s) compared to those without previous malignancy (mean = 4.15 vs 3.30, *p* < 0.001), but only a trend towards a higher perceived chance of developing PC (mean = 4.45 vs 4.11, *p* = 0.055). There was a strong significant correlation in *BRCA2* carriers between personal cancer history and perceived chance of PC (r = 0.67, *p* = 0.001, *n* = 22) and other cancer(s) (r = 0.69, p < 0.001, n = 22). Additional statistical analyses for perceived chance of PC and other cancer(s) are shown in Table 3. Interestingly, there were no significant correlations between participants’ perceived chance of PC and the objective 5-year PC risk (r = − 0.17, *p* = 0.128), lifetime PC risk (r = 0.19, *p* = 0.091), and probability of a PC susceptibility gene (r = 0.21, *p* = 0.067) as calculated by PancPRO.

**Table 3:** Analyses of perceived chance of PC and other cancer(s)

Entire cohort (*n* = 102)		
Differences (mean)	Perceived chance PC	Perceived chance other cancer(s)
Male	4.13 (95% CI 3.87–4.39)	3.50 (95% CI 3.16–3.84)
Female	4.20 (95% CI 4.01–4.39)	3.46 (95% CI 3.23–3.69)
*p*-value	0.564	0.962
Depression	3.94 (95% CI 3.48–4.4)	3.29 (95% CI 2.89–3.69)
No depression	4.22 (95% CI 4.06–4.38)	3.51 (95% CI 3.30–3.72)
*p*-value	0.272	0.368
Correlations		
Age at enrolment r	−0.21	− 0.27
*p*-value	0.833	0.787
Affected FDR r	−0.09	–
*p*-value	0.381	–
Affected FDR/SDR r	−0.02	–
*p*-value	0.816	–
FPC cohort (n = 79)		
Correlations		
Affected FDR r	−0.09	−0.19
*p*-value	0.445	0.094
Affected FDR/SDR r	0.09	−0.04
p-value	0.435	0.703
5-year PC risk r	−0.17	–
*p*-value	0.128	–
Lifetime PC risk r	0.19	–
*p*-value	0.091	–
Probability of PC susceptibility gene r	0.21	–
*p*-value	0.067	–

### Genetic counselling experience

Eighty-eight participants returned the 1-month questionnaire (86.3% response rate) but only 64 participants (73%) responded to all statements, resulting in variability in response numbers. Overall, participants provided positive feedback regarding their genetic counselling. Most (89%, *n* = 77) wanted to undergo genetic testing to clarify their PC risk, two-thirds (*n* = 54) thought current genetic testing would help them, and of those tested, 91% (*n* = 48) were glad they had testing. Participants reported utility of counselling even without genetic testing, with 61% (*n* = 52) strongly disagreeing that genetic counselling should be delayed until more genes are identified, and 68% (*n* = 58) wanted another appointment when more information was available. Overall, 79% (*n* = 67) found the genetic counselling appointment to be helpful, and 88% (*n* = 75) reported they would recommend counselling to their relatives.

Free text responses describing the “most useful” and “least useful” aspects of genetic counselling were further categorised into main themes, with illustrative quotations provided (Table 4 and 5). Fifty-seven participants (65%) included free-text responses about the most useful part of genetic counselling, with reported benefits including: gaining knowledge and understanding of PC risk; feeling empowered to proactively monitor PC risk; receiving support; and the potential to help future generations.

**Table 4:** Illustrative comments of participants’ response to genetic counselling received

Most useful aspect of genetic counselling	
1. Increase knowledge, gain information and understanding	
• “Knowing my risks and having them explained in a straight-forward manner. Knowledge gives you the power to deal with the situation” (P3356 – *BRCA2* carrier)	
• “Having someone to call with questions, someone to turn to for information. Easy access to counsellors was much appreciated” (P4045 – Not tested)	
• “Gave me more info about FPC than I was aware of earlier” (P3652 – No mutation identified)	
2. Risk assessment and genetic testing	
• “Getting a written risk assessment” (P3475 – Not tested)	
• “Finding out about risk factors for PC and understanding likelihood of cancer in my family being genetic” (P3103 – Not tested)	
• “Absence of *BRCA1/2* mutations meant decreased potential risk for my daughters” (P3832 – No mutation identified)	
3. Psychosocial benefits (e.g. reassurance)	
• “Increased my confidence that more research is being conducted to develop cures. Being part of the program enables screening and hopefully early detection in the event I contract the disease” (P4122 – *BRCA2* carrier)	
• “Exploring explicitly my possible concerns or reactions” (P3920 – No mutation identified)	
• “Talking about my problems with an empathetic expert is always a positive” (P4056 – No mutation identified)	
4. Benefit to other family members/society motivated research participation	
• “We might find the [PC] gene. Although it may not help me, it might help my children and others. I know where the testing is up to, and I am informed” (P3126 - No mutation identified)	
• “Useful for those doing the research” (P4024 – Not tested)	
• “Reassurance that research is continuing to prevent/cure PC” (P3139 – No mutation identified)	
Least useful aspect of genetic counselling	
1. Not specific to PC	
• “As a definitive genetic link is yet to be found, a clear risk factor cannot yet be given” (P4139 – Not tested)	
• “I was not given any info regarding PC” (P3954 – Not tested)	
2. Inadequate information provision	
• “Counselling was a complete waste of time - nothing was discussed concerning cancer. Lack of coordination, no written summary received” (P3146 – Not tested)	
• “The risk wasn’t clearly explained at the session” (P3475 - No mutation identified)	
• “Not a useful session. The genetic basis of familial pancreatic cancer is unknown” (P4018 – Not tested)	
3. Limitations of “counselling”	
• “After caring for 2 family members with pancreatic cancer, and watching another, I don’t know if counselling helped me” (P3127 – No mutation identified)	
• “The worry caused after the initial consultation” (P3942 – *BRCA2* carrier)

**Table 5:** Response to statements about the genetic counselling experience as part of the PC screening program

Statement	Strongly agree n (%)	Partially agree n (%)	Neutral n (%)	Partially disagree n (%)	Strongly disagree n (%)	Total n (%)
I knew beforehand that I would receive genetic counselling as part of my research visit	**65 (75.6)**	11 (12.8)	8 (9.3)	1 (1.2)	1 (1.2)	86 (100)
Before genetic counselling, I had already read or heard a fair amount about hereditary pancreas cancer	**36 (41.9)**	30 (34.9)	9 (10.5)	6 (7.0)	5 (5.8)	86 (100)
The genetic counselling session was helpful to me	**46 (54.1)**	21 (24.7)	10 (11.8)	4 (4.7)	4 (4.7)	85 (100)
I would have preferred to have only the endoscopy procedure and not genetic counselling	8 (9.4)	4 (4.7)	23 (27.1)	8 (9.4)	**42 (49.4)**	85 (100)
I would have preferred more information about hereditary pancreas cancer	12 (13.8)	21 (24.1)	**31 (35.6)**	8 (9.2)	15 (17.2)	87 (100)
Scientists do not currently know enough about hereditary pancreas cancer to help me	11 (12.6)	22 (25.3)	**32 (36.8)**	14 (16.1)	8 (9.2)	87 (100)
I would recommend genetic counselling for pancreas cancer to a friend or relative with a family history of pancreas cancer	**65 (76.5)**	10 (11.8)	6 (7.1)	1 (1.2)	3 (3.5)	85 (100)
The genetic information was too complex	1 (1.2)	7 (8.4)	23 (27.7)	18 (21.7)	**34 (41.0)**	83 (100)
The written summary of the visit was useful	**36 (48.6)**	17 (23.0)	16 (21.6)	0 (0)	5 (6.8)	74 (100)
I will share the written summary with my family members	**46 (63.0)**	12 (16.4)	8 (11.0)	3 (4.1)	4 (5.5)	73 (100)
I think genetic counselling for pancreas cancer is helpful, even if the “pancreas cancer gene” has not been found	**55 (66.3)**	15 (18.1)	10 (12.0)	1 (1.2)	2 (2.4)	83 (100)
If the “pancreas cancer gene” were found, I would want to be tested for it	**78 (88.6)**	6 (6.8)	3 (3.4)	0 (0)	1 (1.1)	88 (100)
I do not think genetic counselling should be offered for pancreas cancer until the “pancreas cancer gene” has been found	3 (3.5)	2 (2.3)	14 (16.3)	15 (17.4)	**52 (60.5)**	86 (100)
I would be interested in another genetic counselling session when more information is learned about pancreas cancer	**58 (68.2)**	18 (21.2)	5 (5.9)	1 (1.2)	3 (3.5)	85 (100)
I think that the pancreas cancer in my family is caused by a gene mutation	**37 (43.0)**	13 (15.1)	34 (39.5)	1 (1.2)	1 (1.2)	86 (100)
I think I inherited a gene mutation that causes pancreas cancer	23 (27.7)	11 (13.3)	**45 (54.2)**	2 (2.4)	2 (2.4)	83 (100)
(for participants who have NOT previously had genetic testing related to pancreas cancer): Even though the “pancreas cancer gene” has not been found, I still want to get genetic testing for one or more of the syndromes discussed during genetic counselling	**28 (70.0)**	4 (10.0)	6 (15.0)	1 (2.5)	1 (2.5)	40 (100)
(for participants who HAVE previously had genetic testing related to pancreas cancer): I am glad that I had genetic testing for cancer risk for one or more of the syndromes discussed during genetic counselling	**44 (83.0)**	4 (7.5)	5 (9.4)	0 (0)	0 (0)	53 (100)
I do not feel that current genetic testing is likely to help me	5 (6.1)	3 (3.7)	20 (24.4)	9 (11.0)	**45 (54.9)**	82 (100)

^a^The most frequent response is shown in bold

In contrast, only 14 participants (16%, *n* = 9 not tested, *n* = 4 no mutation identified, *n* = 1 *BRCA2* carrier) provided comments about the least useful part of genetic counselling. Of these, 10 comments (71%) reflected inadequate information provision, limited understanding of PC susceptibility genes and/or ability to estimate an individual’s PC risk, and insufficient follow-up. Twelve participants (14%) reported they did not receive a written summary. This included four siblings who attended a one-off group genetic counselling session, to accommodate for interstate travel and to enable attendance for their baseline EUS together. Additionally, one *BRCA2* carrier indicated that counselling induced worry and one FPC participant commented that counselling does not resolve the grief caused by losing multiple family members.

## Discussion

We evaluate the role of genetic counselling and testing in a national high-risk PC screening cohort and assess the utility of PancPRO to provide a personalised PC risk assessment. We also provide invaluable insights into the recruitment and management of the Australian PC screening program, including barriers to screening uptake. Due to the high mortality of PC and uncertain benefit of screening at this stage [37], the genetic counselling needs of these high-risk individuals may differ from those with other hereditary cancer syndromes and data obtained from our cohort provide important insights in this area. Through the development of formal genetic counselling guidelines, we describe our genetic counselling recommendations for HRI, which in combination with participant feedback, are hoped to guide practice improvements.

### Recruiting high-risk PC individuals

Establishing a national registry to capture PC kindreds was a successful approach to target HRI, with over 66% of the enquiries received via the AFPaCC website. Once initial contact was made, additional at-risk relatives were identified and invited to join the registry and/or PC screening program (*n* = 134). Moreover, the registry enables easy identification and contact of individuals who may become eligible over time (i.e. with age; an incident PC diagnosis in the family). Although clear inclusion criteria were available online, approximately half of those who made contact had only 1FDR with PC, and it was necessary for the clinical research coordinator to provide personalised clarification around sporadic and familial PC. Referrals from FCC identified eligible and motivated individuals who were ready to attend their baseline EUS, resulting in a streamlined recruitment process.

Nearly half of the eligible individuals who expressed interest in screening, chose not to pursue participation. Accessibility (e.g. financial and logistical challenges of travel), time constraints and the amount of paperwork required were reported as the main barriers for at-risk individuals. The Australian population is widely dispersed across a large land mass, and participants must self-fund transport and/or accommodation. These reported barriers align with a German PC screening study, which found the cost of travel and the time required for participation as factors impacting screening uptake [38]. Many eligible individuals did not actively decline participation, suggesting screening uptake may increase should the perceived benefits improve and the perceived or actual barriers to participation be overcome. Strategies to address these challenges include: expansion of the screening program into other states with concomitant funding for a full-time national coordinator, funding for travel arrangements, subsidised local accommodation and simplification of the questionnaire.

### Genetic counselling experience

Participants believed genetic counselling to be helpful even without genetic testing, would recommend it and were interested in genetics follow-up as more PC genes are discovered. Participants felt strongly that their family history was due to a pathogenic variant, though were split on whether they had inherited it. The current study reflects the increasing expectation that genetic testing will clarify an individual’s PC predisposition. Ninety-one percent of our tested participants were glad to have undergone testing and two-thirds felt that current genetic testing would help them, compared to approximately 40% previously reported [39]. Key differences between our cohort and that of Axilbund et al. [39], were 1) genetic counselling in our study was conducted by multiple genetic counsellors (employed in numerous services across four Australian states), compared to a single genetic counsellor, and 2) our cohort contained individuals with variable family histories, whilst participants in the earlier study had at least three affected relatives. These data suggest that the perceived benefits of genetic counselling are not influenced by patients having FPC or a known pathogenic variant.

Our participants reported that genetic counselling provided essential information to aid understanding of risks (both inherited and environmental) and was empowering. This correlates with previous reports on patient perception following genetic counselling for PC, which indicate improved understanding and psychological function [27, 28]. Consistent with Underhill et al. [37], participants were motivated to prevent PC mortality and felt reassured by PC surveillance. Disappointment with genetic counselling was present in a minority, with comments reflecting distress at the limitations in current knowledge of PC predisposition. Some participants indicated that their counselling focused on their *BRCA2* carrier status, rather than providing information regarding their PC risk (understandable given almost two-thirds of the *BRCA2* cohort were female, and nine had a personal history of breast cancer). Emphasizing the PC risk associated with hereditary breast and ovarian cancer (HBOC) and Lynch syndrome is not appropriate for all families, as the incidence of PC is lower in these syndromes [40, 41] though should be tailored for those with a family history of PC. As all *BRCA2* carriers completed counselling off-protocol, some many years prior to the screening program enrolment, it is conceivable that some participants received counselling prior to their relatives’ diagnosis with PC. New screening participants who previously received genetic counselling, may therefore benefit from a review appointment to obtain current information on PC screening, risk management and genetic testing options.

Several participants commented that they did not receive a written summary of their counselling appointment(s), despite it being common genetic counselling practice [42]. Written summaries are reported to reduce cancer anxiety and improve accuracy in risk perception [25], assist patient understanding and recall, and facilitate accurate family communication [26, 43] Importantly, participants in the current study who received summaries, found them easy to understand and almost 80% either partially or strongly agreed that they would share it with relatives. These data suggest that documenting counselling discussions for PC is important due to current limitations with informative genetic testing, accurate risk assessments and a need to individualise recommendations based on family history and environmental risk factors.

Participants with the highest perceived chance of developing other cancer(s), were either *BRCA2* carriers, had a personal history of cancer, or a family history of multiple cancer types, consistent with results from Rantala et al. [24]. Our data suggest these participants may be most vulnerable to increased cancer worry and perceived risk, which may warrant exploration during counselling. Genetic testing where appropriate, might help clarify an individual’s cancer susceptibility, may alleviate worry of developing other cancer(s) and contribute to better psychological outcomes. Participants’ interest in receiving additional counselling further demonstrates the importance of establishing a plan for genetics review to facilitate genetic testing as more information about FPC becomes available.

Although these data support the value of genetic counselling despite current limitations in testing utility, the genomic era will likely transform genetic counselling practices for FPC [44]. Studies are demonstrating that family history alone is a poor predictor of mutation status [45, 46] and further genomic characterisation of genetic modifiers in PC will improve understanding of FPC and positively impact genetic counselling processes [44]. Elucidation of causative pathogenic variants may further clarify the cancer risk(s) for unaffected blood relatives and assist appropriate stratification into high-risk screening programs [47].

### Utility of PancPRO for FPC risk-assessment

PancPRO is not commonly used in Australia during the provision of genetic counselling. Using hypothetical scenarios, Leonardi et al. [35] found PancPRO risk estimates to be a valid method to stratify high-risk FPC families to PC screening protocols. Our study prospectively assesses the utility of PancPRO to provide personalised PC risk estimates in a true cohort of participants. Overall, our mean lifetime PC risk of 10.17% is consistent with that reported by Barnes et al. [34], the only other study to generate PancPRO risk estimates for FPC screening participants. Further comparison is not possible, as their cohort consisted of only 33 individuals and no subgroup analysis was published. The mean PancPRO lifetime PC risk values calculated for our participants with two affected FDR was 9.5%, which is comparable to those reported following prospective analysis of incident pancreatic cancers from the National Familial Pancreatic Tumor Registry (lifetime risk 8–12%) [10, 11]. However, the lifetime PC risk previously reported for those with ≥3FDR (lifetime risk 40%) [10, 11] were higher than the calculated PancPRO lifetime risks for our cohort (9.7%). Potentially, this is due to the small number of participants with 3FDR (*n* = 11) and it must be noted that 2/11 participants with at least 3FDR had both parents and a sibling affected. PancPRO reported a large range in lifetime PC risk estimates for participants with 1FDR (3.7–14.4%), yet the overall mean was equivalent to the 2FDR and 3FDR subgroups. This is likely due to the variable family history and age of diagnosis in these subgroups, or the unreliability of PancPRO. Further studies with subgroup analysis are needed to clarify this observed issue.

Incorporation of pathogenic variant status and environmental risk factors in future versions of PancPRO (or alternate PC risk estimation programs), once reliable PC penetrance data exist would be beneficial. Due to the genetic heterogeneity of PC, developing a comprehensive risk estimation model would aid genetic counselling and facilitate informed decision-making regarding risk-modification behaviours. Personalised risk assessments may also improve the uptake of screening for the subset of eligible participants who failed to see benefit.

### Study limitations

Inherited forms of PC are rare and recruitment of HRI is challenging and time-consuming. Participants in our high-risk PC cohort had variable personal and family history of cancer, and the cohort is biased toward younger, healthy participants, resulting in low 5-year PC risk estimates. More detailed information about barriers to screening uptake would have been valuable. Another limitation is the lack of histopathologically confirmed pancreatic malignancies in all probands, with reliance on self-reports and death certificates for some kindreds; however, Fiederling et al. [48] found self-reported PC family histories are valid for preventative counselling.

As participants received genetic counselling at different genetics services and were recruited across a period of six years, the information provided and genetic testing practices may have varied. Although the genetic counselling guidelines were circulated to improve consistency, some completed counselling several years prior to screening program recruitment. Their views on the genetic counselling process are likely to exhibit recall bias due to the greater time since counselling occurred.

## Conclusions and future directions

This study is the first to describe results from the Australian pancreatic cancer screening program. Participants reported positive genetic counselling experiences with feedback indicating improved knowledge and feelings of empowerment. Our data indicate that participants want genetic testing and clarification of their mutation status in relation to PC predisposition. Tailored discussion of PC risk in patients with *BRCA2* mutations, provision of a written summary in all cases and setting a plan for genetics review were identified as areas for improvement. Updating the genetic counselling guidelines to include participant feedback may further support exploration of participants’ perceived cancer chance within the context of their estimated risk and improved risk communication strategies. PancPRO has the potential to become a useful, personalised risk assessment and communication tool for FPC for use in clinical practice. We highlight that extension of PancPRO to model PC risk for individuals with pathogenic variants and incorporation of environmental factors would provide greater clarity regarding individualised PC risk and its’ development should be a research priority.

Strategies to overcome barriers to recruitment in Australia to improve program accessibility are needed. We aim to establish a uniform screening program across Australia, provide consistent genetic testing and counselling and facilitate a centralised, national database to promote efficient access for PC researchers and close international collaborations.

## Supplementary information

**Additional file 1.** Inclusion and exclusion criteria. (DOCX 17 kb)

**Additional file 2.** Genetic counselling guidelines. (DOCX 31 kb)

## Data Availability

The dataset supporting the conclusions of this article is included within the article and its additional files.

## Abbreviations

AFPaCC: Australian Familial Pancreatic Cancer Cohort
APGI: Australian Pancreatic Cancer Genome Initiative
CAPS: Cancer of the Pancreas Screening
CT: Computed tomography
EUS: Endoscopic ultrasound
FCC: Familial cancer clinic
FDR: First degree relative
FPC: Familial pancreatic cancer
GP: General Practitioner
HBOC: Hereditary Breast and Ovarian Cancer
HRI: High-risk individuals
MIC-1: Macrophage inhibitory cytokine 1
MRI: Magnetic resonance imaging
PC: Pancreatic cancer
PJS: Peutz-Jeghers syndrome
SDR: Second degree relative
STVH: St Vincent’s Hospital
VUS: variant of uncertain significance
