# Supplementary material for: Genetic counselling and personalised risk assessment in the Australian pancreatic cancer screening program
Source: Hered Cancer Clin Pract. 2019 Oct 23;17:30. doi: 10.1186/s13053-019-0129-1 (PMC6813120; doi:10.1186/s13053-019-0129-1)
Supplement: Supplementary file 1 — Additional file 1. Inclusion and exclusion criteria. (DOCX 17 kb) [file 13053_2019_129_MOESM1_ESM.docx]

**Appendix 1. Inclusion and exclusion criteria**

Pancreatic Cancer Screening Program

**INCLUSION CRITERIA**

High Risk Group 1: Familial Pancreatic Cancer

1. Aged 50-80 years (or 10 years younger than the youngest relative with PC, AND
2. Member of a family with 2 or more blood relatives with PC on the same side of the family. If only 2 family members are affected, both must be an FDR of the individual being screened. If there are ≥3 affected family members, at least one must be an FDR of the individual being screened.

High Risk Group 2: Peutz-Jeghers Syndrome

1. Age > 30 years old and < 80 years old, AND
2. Clinical diagnosis of Peutz-Jeghers Syndrome or carrier of a germline *STK11* pathogenic variant.

High Risk Group 3: BRCA2 pathogenic variant carriers

1. Age > 40 years old and < 80 years old (or 10 years younger than the youngest relative with PC) AND
2. Patient is a carrier of a *BRCA2* pathogenic variant AND
3. There is ≥1 pancreatic cancer in the family (FDR or SDR, confirmed or likely carrier of the pathogenic variant)

High Risk Group 4: Hereditary Pancreatitis

1. Age > 40 years old and < 80 years old (or 10 years younger than the youngest relative with PC) AND
2. Previous diagnosis of Hereditary Pancreatitis or known carrier of a *PRSS1* or *SPINK1* pathogenic variant.

High Risk Group 5: PALB2 gene carrier*

1. Age > 50 years old and < 80 years old (or 10 years younger than the youngest relative with PC) AND
2. Patient is a carrier of a PALB2 pathogenic variant AND
3. There is ≥1 pancreatic cancer in the family (FDR or SDR, confirmed or likely carrier of the pathogenic variant)

High Risk Group 6: Lynch syndrome mutation carrier /hereditary non polyposis colorectal cancer mutation carrier ( MLH1, PMS2, MSH6, MSH2 mutation ) *

1. Age > 50 years old and < 80 years old (or 10 years younger than the youngest relative with PC) AND
2. Patient is a Lynch syndrome mutation carrier AND
3. There is a ≥1 FDR with pancreatic cancer

High Risk Group 7: Familial Atypical Multiple Melanoma Moles (FAMMM) syndrome (CDKN2A/p16 mutation carrier)*

1. Age > 50 years old and < 80 years old (or 10 years younger than the youngest relative with PC) AND
2. Patient is a carrier of p16/ CDKN2A pathogenic variant

**EXCLUSION CRITERIA**

Patients will be excluded if they have any of the following:

1. personal history of pancreatic cancer or previous pancreatic surgery

2. medical illnesses that increase the risk of endoscopy and possible surgery: unstable angina,

severe congestive heart failure requiring daily medication, severe chronic obstructive

pulmonary disease (COPD) requiring daily medication, pulmonary hypertension, obstructive

sleep apnoea requiring treatment with BIPAP or CPAP

3. history of severe chronic kidney disease with an estimated glomerulofiltration rate (eGFR)

< 30 ml/min, acute renal failure, cirrhosis of the liver, or chronic active hepatitis

4 poor performance status Karnosfky performance status of < 60

5. subjects will not be enrolled if they feel that they would not be interested in treatment of

pancreatic abnormalities found during this study, such as possible pancreas surgery

6. bleeding diathesis (clotting problems) or a history of thrombocytopenia (low platelet

count)

7. previous gastric or biliary surgery other than cholecystectomy

8. cancer (other than skin cancer) within last 5 years not in remission

9. history of AIDS

10. inability to provide informed consent

11. pregnancy

12. morbid obesity with body mass index (BMI) >35

13. dementia.
