# Supplementary material for: Genetic counselling and personalised risk assessment in the Australian pancreatic cancer screening program
Source: Hered Cancer Clin Pract. 2019 Oct 23;17:30. doi: 10.1186/s13053-019-0129-1 (PMC6813120; doi:10.1186/s13053-019-0129-1)
Supplement: Supplementary file 2 — Additional file 2. Genetic counselling guidelines. (DOCX 31 kb) [file 13053_2019_129_MOESM2_ESM.docx]

**Appendix 2. Genetic counselling guidelines**

**SCREENING FOR PANCREATIC CANCER IN HIGH RISK INDIVIDUALS:**

**Genetic Counselling Guidelines and Considerations**

These guidelines relate to the Screening for Pancreatic Cancer in High-Risk Individuals clinical research trial being carried out at St Vincent’s Hospital, Sydney and Austin Health in Melbourne, in conjunction with the Australian Familial Pancreatic Cancer Cohort (AFPaCC) registry at the Garvan Institute of Medical Research.

It is anticipated that eligible participants may be seen in the Familial Cancer Clinics (FCC’s) via 2 mechanisms, being:

- Direct referral from the screening gastroenterologists, as a genetic counselling session is included in the research protocol
- Coincidentally through another referral, such as *BRCA2* testing, or a family already known to the FCC with a strong family history of pancreatic cancer
- Referral from AFPaCC registry

The purpose of this document is to highlight current literature in familial pancreatic cancer, and to facilitate consistent discussions with those considered high risk across Australia.

1. **Purpose of counselling session:**
2. If a participant is already enrolled in the screening trial, and attending genetic counselling as per the protocol, the main aims of the session would be to:
   - clarify and verify family history
   - discuss individual risks
   - discuss any relevant genetic testing
   - explore any psychosocial issues related to participation
   - discuss any lifestyle changes that can be made to reduce personal risks (obesity, smoking, alcohol )
3. If a patient is being seen in the FCC for another referral, and are recognised to be eligible for the screening trial, the main aims would be:
   - discuss risks (if appropriate)
   - introduce the familial pancreatic cancer registry (AFPaCC) and the screening trial, if appropriate (Appendix 1: Screening Trial Inclusion Criteria)
   - provide contact details for research coordinators

*Note: It is the role of the specialist gastroenterologists to outline benefits and limitations of being involved in the screening trial with each participant and to consent participants*

1. **Key points to discuss:**
   - The majority (~90%) of pancreatic cancer (PC) cases are considered sporadic
   - PC is relatively uncommon, accounting for 2.2% of all cancers in Australia (e.g. 2, 546 people diagnosed in 2009) ^i^
   - Current risk in Australia by 75yrs is 1 in 141 (0.71%). The risk increases to 1 in 68 (1.47%) by 85yrs ^1^
   - Males are at a slightly higher risk, with a Male:Female ratio of 1.3:1. The average age at diagnosis is 70yrs.

Below are some definitions and statistics that may assist when introducing the concept of sporadic and familial pancreatic cancer.

Sporadic pancreatic cancer

• Increased risk has been associated with cigarette smoking, increased body mass index, increased age, heavy alcohol consumption, a diagnosis of diabetes mellitus and a family history of pancreatic cancer

Familial pancreatic cancer (FPC**)**

- FPC accounts for approximately 5-10% of all cases of pancreatic cancer
- Defined as **at least one pair of first-degree relatives** (FDR) affected by pancreatic cancer, with the suggestion of an autosomal dominant trait and incomplete penetrance.
- The genetic cause of FPC remains largely unknown, although *BRCA2* has been found to be positive in 6-16% of individuals with FPC ^2^ and mutations in *PALB2* explain 1-3% of FPC kindreds.
- Screening is recommended for individuals considered to be at a high risk of developing pancreatic cancer (i.e. >5% lifetime risk, or fivefold increased relative risk) although there is no consensus on age to commence screening.

| **Family History of PC** | **Relative risk of PC** | **Cumulative life time risk of PC %** |
| --- | --- | --- |
| General population | 1.0 | 1.3% |
| 1 first degree relative | 1.8-2.7 | 4% |
| 2 first degree relatives | 6.4 | 8-12% |
| 3 first degree relatives | 32 | 16-38% |

*† Statistics from the US National Familial Pancreatic Tumour Registry (NFPTR) at John Hopkins University ^3,4^*

- Age of onset is important. The risk is higher if there is an FDR with onset of PC younger than 50 years of age.

For example, the lifetime risk of PC in an individual with 3 FDR is 38% if one first-degree relative had cancer at age 40 compared with 19% when the youngest age of onset of cancer in the family is 60 ^5^

- Evidence suggests that smoking increases the risk of pancreatic cancer by 2-3.7 times over the inherited predisposition and lowers the age of onset by 10 years ^6^

Inherited pancreatic cancer syndromes

• Known genetic syndromes that increase the risk of pancreatic cancer ^7,8^

| **Cancer Syndrome** | | **Genes** | **Relative risk of PC** | **Cumulative life time risk of PC** |
| --- | --- | --- | --- | --- |
| Hereditary Pancreatitis ^9^ | | *PRSS1,*  *SPINK1* | 53-80 | 40% |
| Peutz-Jeghers Syndrome ^10^ | | *STK11* | 132 | 36% |
| Hereditary Breast and Ovarian Cancer ^11*^ | | *BRCA2* | 3.5 – 5.9 | 5% |
| Familial Atypical Multiple Mole-Melanoma (FAMMM) ^12 #^ | | *CDKN2A/p16* | 13-22 | 10-18% |
| Lynch Syndrome ^13^ | | *MSH2, MLH1, MSH6, PMS2* | 8.6 | 4% |
|  |  | |  |  |
| ** The higher range of risk is expected when other risk factors are included, such ≥1 FDR with pancreatic cancer, male gender, cigarette smoking*  *# There is no evidence in Australia to support increased risk in FAMMM* | | | | |

1. **Psychosocial considerations**
2. Perceived risk^14^
   - Individuals with a multiple family member affected by PC may have higher levels of perceived risk and worry regarding PC
   - Elevated levels of concern may explain high compliance rates for PC screening in international studies (67-95%)
   - Studies have shown that participating in screening does not increase levels of concern/worry^15^

*Note: It would be important to reiterate difference between relative and absolute risks at this stage*

1. Resistance to Genetic Counselling
   - Some participants may be initially resistant to genetic counselling, especially if they are aware of the current gaps in familial pancreatic information, and lack of causative gene

*Note: Studies have shown participants found genetic counselling useful in FPC, at least to further understand inheritance patterns and the various cancer syndromes associate with pancreatic cancer and would seek genetic testing if available* ^16^

1. Motivation to Participate ^17^

Individuals may be driven to participate in pancreatic cancer screening trials due to a number of reasons, which can include:

- - The belief that early detection of pancreatic cancer may result in a higher cure rate (as at this stage symptomatic cancer is often not curable)
  - Obtaining a sense of control over their own body and health
  - Contributing to scientific research
  - Following recommendations, or referral, by physician
  - Feeling obligated, or pressured by other family members

*Note: any concern regarding an individual’s motivation to be involved in the screening trial (e.g. pressure from relatives) should be discussed with the specialist gastroenterologists.*

*Participation is completely voluntary, and participants can withdraw at any time without any consequence to their ongoing care.*

- - The health belief model (HBM) is a theoretical framework to explain cancer screening uptake in asymptomatic individuals.
  - 5 key factors include: Perceived susceptibility; perceived severity; perceived benefits of screening; perceived barriers of screening; self-efficacy in screening up-take ^18^

1. Resistance to Participate

Possible concerns about participation in the screening program may include:

- - Inconvenience of having endoscopic ultrasounds at St Vincent’s Hospital, Sydney
  - Perceived discomfort of procedure
  - Travel costs of being involved, especially for rural and interstate participants

*Note: These concerns should have been addressed prior to referral to the Family Cancer Clinic. Please notify the specialist gastroenterologists involved in the study if you feel the participant having reservations at this stage of the protocol.*

**Australian Pancreatic Screening in High Risk Individuals: Inclusion & Exclusion Criteria**

**1. Familial Pancreatic Cancer**

Age ≥50 years old (or 10 years younger than the age of youngest relative with pancreatic cancer) and <80 years old, AND

Member of family with ≥ 2 blood relatives with a history of pathologically proven pancreatic cancer

• If only 2 family members are affected, then both must have a first-degree relationship with each other and the individual being screened (e.g parent-sibling or 2 siblings)

• If 3 or more family members affected, than at least one must have a first-degree relationship with the person being screened

**2. Peutz-Jeghers Syndrome (PJS)**

Age >30 years old and <80 years old, AND

Clinical diagnosis of PJS or proven carrier of *STK11* mutation

**3. BRCA2 Mutation Carriers**

Age ≥40 years old (or 10 years younger than the age of youngest relative with pancreatic cancer) and <80 years old, AND proven carrier of a known pathogenic BRCA2 mutation, AND at least one pancreatic cancer in the family (FDR or SDR, confirmed or likely carrier of the pathogenic variant)

**4. Hereditary Pancreatitis (HP)**

Age ≥40 years old (or 10 years younger than the age of youngest relative with pancreatic cancer) and <80 years old, AND clinical diagnosis of HP or proven carrier of *SPINK1* mutation

**5. PALB2 mutation carrier**

Age > 50 years old and < 80 years old (or 10 years younger than the youngest relative with PC) AND carrier of a PALB2 pathogenic variant and at least one pancreatic cancer in the family (FDR or SDR, confirmed or likely carrier of the pathogenic variant)

**6. Lynch syndrome mutation carrier /hereditary non polyposis colorectal cancer mutation carrier ( MLH1, PMS2, MSH6, MSH2 mutation )**

Age > 50 years old and < 80 years old (or 10 years younger than the youngest relative with PC) AND patient is a Lynch syndrome mutation carrier AND one FDR with pancreatic cancer

**7. Familial Atypical Multiple Melanoma Moles (FAMMM) syndrome (CDKN2A/p16 mutation carrier)**

Age > 50 years old and < 80 years old (or 10 years younger than the youngest relative with PC) AND

patient is a carrier of p16/ CDKN2A pathogenic variant

**Additional requirements for all eligible high-risk participants:**

• All persons with known genetic mutation must have proof of mutation status.

• A good faith attempt will be made to confirm pancreatic cancers in the family members using medical records, histopathology reports or death certificate.

**NOTE**

Other PC predisposition genes (*BRCA1*, *ATM*, *TP53* and *APC*) and are not currently included in Australian screening program. . Recent studies suggesting *BRCA1* is a moderate risk PC gene ^19^ supports our decision to exclude patients with *BRCA1* pathogenic variants from high-risk screening, despite the inclusion of *BRCA1* carriers in other screening protocols ^20,21^. Evidence supporting *ATM* as a high-risk PC predisposition gene is accumulating ^22,23^. and inclusion of *ATM* in our program is under review.

REFRENCES

1.Australian Institute of Health and Welfare (AIHW) 2012. ACIM (Australian Cancer Incidence and Mortality) Books. AIHW: Canberra

2. Couch FJ, Johnson MR, Rabe KG, Brune K, de Andrade M, Goggins M, Rothenmund H, Gallinger S, Klein A, Petersen GM, Hruban RH. The prevalence of BRCA2 mutations in familial pancreatic cancer. Cancer Epidemiol Biomarkers Prev 2007;16:342-346

3. Klein AP, Brune KA, Petersen GM, et al. Prospective risk of pancreatic cancer in familial pancreatic cancer kindreds. Cancer Research 2004;64:2634-2638

4. Tersmette AC, Petersen GM, Offerhaus GJ, Falatko FC, Brune KA, Goggins M, Rozenblum E, Wilentz RE, Yeo CJ, Cameron JL, Kern SE, Hruban RH. Increased risk of incident pancreatic cancer among first-degree relatives of patients with familial pancreatic cancer. Clin Cancer Res 2001;7:738-744

5. Brune KA, Lau B, Palmisano E, Canto M, Goggins MG, Hruban RH, Klein AP. Importance of age of onset in pancreatic cancer kindreds. J Natl Cancer Inst 2010;102:119-126

6. Rulyak SJ, Lowenfels AB, Maisonneuve P, Brentnall TA. Risk factors for the development of pancreatic cancer in familial pancreatic cancer kindreds. Gastroenterology 2003;124:292-299

7. Hruban RH, Canto M, Goggins M, Schulick R, Klein AP. Update on familial pancreatic cancer. Adv Surg 2010;44:293-311

8. Klein AP. Genetic susceptibility to pancreatic cancer. Molecular Carcinogenesis 2012;51:14-24

9. Lowenfels AB, Maisonneuve P, DiMagno EP, Elitsur Gates LK Jr, Perrault J, Whitcomb DC. Hereditary pancreatitis and the risk of pancreatic cancer. International Hereditary Pancreatitis Study Group. J Natl Cancer Inst 1997;89:442-446

10. Giardiello FM, Brensinger JD, Tersmette AC, Goodman SN, Petersen GM, Booker SV, Cruz-Correa M, Offerhaus JA. Very high risk of cancer in familial Peutz-Jeghers syndrome. Gastroenterology 2000; 119:1447-1453

11. van Asperen CJ, Brohet RM, Meijers-Heijboer EJ, Hoogerbrugge N, Verhoef S, Vasen HF, Ausems MG, Menko FH, Gomez Garcia EB, Klijn JG, Hogervorst FB, van Houwelingen JC, van’t Veer LJ, Rookus MA, van Leeuwen FE. Cancer risks in BRCA2 families: estimates for sites other than breast and ovary. J Med Genet 2005;42:711-719

12. Goldstein AM, Chan M, Harland M, Hayward NK, et al. Features associated with germline CDKN2A mutations: a GenoMEL study of melanoma-prone families from three continents. J Med Genet 2007;44:99-106

13. Kastrinos F, Mukherjee B, Tayob N, Wang F, Sparr J, Raymond VM, Bandipalliam P, Stoffel EM, Gruber SB, Syngal S. Risk of pancreatic cancer in families with Lynch syndrome. JAMA 2009;302:1790-1795

14. Radecki Breitkopf CM, Sinicrope PS, Rabe KG, et al. Factors influencing receptivity to future screening options for pancreatic cancer in those with and without pancreatic cancer family history. Hereditary Cancer in Clinical Practice 2012;10:8

15. Axilbund JE, Brune KA, Canto MI, et al. Patient perspective on the value of genetic counselling for familial pancreas cancer. Hereditary Cancer in Clinical Practice 2005;3:115-122

16. Maheu C, Vodermaier A, Rothenmund H, et al. Pancreatic cancer risk counselling and screening: impact on perceived risk and psychological functioning. Familial Cancer 2010;9:617-624

17. Harinck F, Nagtegaal T, Kluijt I, et al. Feasability of a pancreatic cancer surveillance program from a psychological point of view. Genetics in Medicine 2011;13:1015-1024

18. Lewis ZK, Frost CJ, Venne VL. Pancreatic cancer surveillance among high-risk populations: Knowledge and intent. Journal of Genetic Counselling 2009;18:229-238

19. Hu, C., H. LaDuca, H. Shimelis, E.C. Polley,F.J. Couch, Multigene Hereditary Cancer Panels Reveal High-Risk Pancreatic Cancer Susceptibility Genes. JCO Precision Oncology, 2018(2): p. 1-28.

18. Canto M.I., J.A. Almario, R.D. Schulick, C.J. Yeo, R.H. Hruban, and M. Goggins, Risk of Neoplastic Progression in Individuals at High Risk for Pancreatic Cancer Undergoing Long-term Surveillance. Gastroenterology, 2018. 155(3): p. 740-751.e2.

21. Konings, I.C.A.W., G.N. Sidharta, F. Harinck, C.M. Aalfs, J.W. Poley, J.M. Kieffer, M.A. Kuenen, E.M.A. Smets, A. Rens, P. Fockens, M.J. Bruno, and E.M.A. Bleiker, Repeated participation in pancreatic cancer surveillance by high‐risk individuals imposes low psychological burden. Psycho‐Oncology, 2016. 25(8): p. 971-978.

22. Shindo, K., J. Yu, M. Suenaga, S., T. Barkley, J. He, M.J. Weiss, C.L. Wolfgang, N.J. Roberts, R.H. Hruban, A.P. Klein, and M. Goggins, Deleterious Germline Mutations in Patients With Apparently Sporadic Pancreatic Adenocarcinoma. J Clin Oncol, 2017. 35(30): p. 3382-3390.

23. Roberts, N.J., Y. Jiao, J. Yu, L. Kopelovich, R.H. Hruban, K.W. Kinzler, and A.P. Klein, ATM mutations in hereditary pancreatic cancer patients. Cancer Discov, 2012. 2(1): p. 41-46.
